# Pharmaco-fUS for Characterizing Drugs for Alzheimer’s Disease – The Case of THN201, a Drug Combination of Donepezil Plus Mefloquine

**DOI:** 10.3389/fnins.2020.00835

**Published:** 2020-08-12

**Authors:** Benjamin Vidal, Marine Droguerre, Marco Valdebenito, Luc Zimmer, Michel Hamon, Franck Mouthon, Mathieu Charvériat

**Affiliations:** ^1^Theranexus, Lyon, France; ^2^Lyon Neuroscience Research Center, Université de Lyon, INSERM, CNRS, Bron, France; ^3^CERMEP-Imagerie du Vivant, Bron, France; ^4^Hospices Civils de Lyon, Lyon, France

**Keywords:** pharmaco-fUS, Alzheimer’s disease, donepezil, mefloquine, connexin

## Abstract

Donepezil is a potent acetylcholinesterase inhibitor, largely used worldwide to alleviate cognitive symptoms in Alzheimer’s disease (AD). Beyond the widely described neuronal impact of donepezil, it was recently shown that targeting connexins, the proteins involved in astrocyte network organization, potentiates donepezil efficacy profile using behavioral tests in AD rodent models. We herein present data demonstrating the potential of functional ultrasound imaging to monitor cerebral activity changes after pharmacological challenge in mice. As an example, we showed that although administration of donepezil or mefloquine alone at low dose had only very limited effects on the signal compared to the baseline, their combination produced marked hemodynamic effects in the hippocampus, in line with previously published behavioral data demonstrating a synergic interaction between both drugs. Thus, the present study provides new perspectives, (i) through the use of pharmaco-fUS, a new non-clinical imaging modality, to move forward drug discovery in AD and (ii) by the profiling of two drug treatments on brain dynamics, one used in AD: donepezil, and the other in development: donepezil combined with mefloquine (THN201) as a modulator of astrocyte network.

## Introduction

Recent evidence suggested that Alzheimer’s disease (AD) not only affects neurons but also non-neuronal cells, and notably astrocytes (Heneka et al., 2015). Astrocytes are glial cells organized in plastic and highly regulated networks. Those networks are constitutively based on transmembrane channels made of connexins, mostly their Cx30 and Cx43 isoforms (Giaume and Liu, 2012; Charvériat et al., 2017). Those proteins are upregulated in AD mice models (Mei et al., 2010) and brains from AD patients (Nagy et al., 1996). Meanwhile, previous non-clinical and clinical studies demonstrated that the modulation of astroglial connexins may enhance efficacy of drugs in neurological disorders (Duchêne et al., 2016; Jeanson et al., 2016; Vodovar et al., 2018; Sauvet et al., 2019). Importantly, we recently demonstrated that mefloquine, a connexin modulator, both used *in vitro* (Picoli et al., 2012, 2019) and *in vivo* (Jeanson et al., 2016; Droguerre et al., 2019), significantly potentiated the efficacy of one of the gold standard treatments in AD, donepezil (Adlimoghaddam et al., 2018), both in scopolamine and amyloid-beta (Aβ) rodent models (Droguerre et al., 2020).

Functional ultrasound (fUS) is a recently developed neuro-imaging modality designed to monitor whole brain activity in specific situations, through the recording of cerebral blood volume (CBV) dynamics (Mace et al., 2011; Urban et al., 2015). Here it has been adapted – further referred to as pharmaco-fUS modality – to assess the hemodynamic effects of two treatments, with donepezil alone versus this drug combined with mefloquine at low dose (combination THN201), in mice. Our study opens interesting perspectives for further evaluation of the central effects of drug treatments, either validated or under development, especially in AD field, using pharmaco-fUS.

## Materials and Methods

All experiments were conducted in strict accordance with the recommendations and guidelines of the European Union (Directive 2010/63/EU) and strictly followed the policies of the French ethic committee for preclinical research. Procedures and protocols herein described were authorized by the French Ministry of Research (authorization reference: APAFIS19829). C57BL/6 male mice (from Janvier Labs, Le-Genest-St-Isle, France) were kept under controlled environmental conditions (22 ± 1°C, 12 h/12 h alternate light/dark cycle, 60% humidity, food and water *ad libitum*) for at least one week before the experiments. Animals were administered intraperitoneally (i.p.) with donepezil 0.25 or 1 mg/kg (DPZ0.25 or DPZ1) alone or combined with mefloquine 1 mg/kg (MEF1).

For pharmaco-fUS imaging, mice were anesthetized with ketamine/medetomidine (70 mg/kg i.p./0.6 mg/kg i.p.) and scanned with a device dedicated to small animal ultrasound neuroimaging (Iconeus, Paris, France). Doppler vascular images were obtained using the Ultrafast Compound Doppler Imaging technique. Each frame was a Compound Plane Wave frame (Montaldo et al., 2009) resulting from the coherent summation of backscattered echoes obtained after successive tilted plane waves emissions. A stack of hundreds of such compounded frames was acquired with very high frame rate. Each transcranial Doppler image was obtained from 200 compounded frames (Montaldo et al., 2009) of Doppler vascular images (Bercoff et al., 2011) acquired at 500 Hz frame rate. Images were acquired every second for 30 min. A fast scan with successive images taken on several coronal planes was performed for positioning the probe in the hippocampus plane (bregma −2 mm).

For each scan, the Allen mouse brain atlas was manually registered on the images and the CBV was extracted from the different regions of interest (ROI). After high-pass filtering and removal of linear trend calculated from the baseline, the signal was expressed as percentage of the baseline between 0 and 10 min.

The voxel-based analysis of fUS data was performed with the software SPM12 (Wellcome Trust Center for Neuroimaging). The images were smoothed using an isotropic Gaussian filter and a first-level analysis was performed on each scan using a general linear model. A second-level analysis was performed at the group level in order to quantify the CBV changes occurring in each condition compared to the vehicle injection by using two-sample *t*-tests. The resulting maps of *T*-scores were converted into *Z*-scores and statistical significance was set at *P* < 0.05.

All data are expressed as mean ± SEM. CBV changes in the different ROI were analyzed by two-way ANOVA followed by Dunnett’s *post hoc* tests. Significance level was set at 0.05.

## Results

**FIGURE 1:**
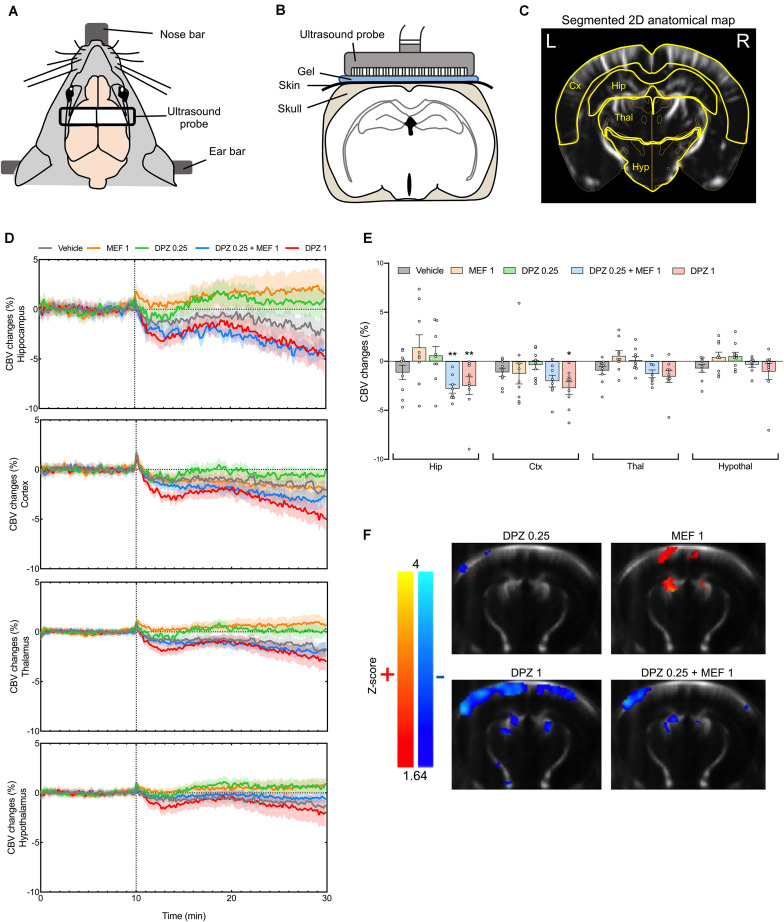
Effects of donepezil and/or mefloquine on cerebral blood volume (CBV) in anesthetized mice after intraperitoneal administration of donepezil 0.25 or 1 mg/kg (DPZ0.25 or DPZ1) alone or combined with mefloquine 1 mg/kg (MEF1). **(A)** Schematic representation of the experimental setup during fUS imaging. **(B)** Position of the fUS probe in a coronal brain plan. **(C)** Typical coronal slice of a mouse brain with segmented regions of interest (yellow) after co-registration of the Allen mouse brain atlas. L, left; R, right. **(D)** Time curves of CBV changes in the different regions of interest (*n* = 9 mice per group, except *n* = 8 mice for DPZ0.25 + MEF1; mean curves ± SEM). The injection time is shown by a dashed line. **(E)** Mean CBV changes during the post-injection period in the different regions of interest in the same animals (means ± SEM). Two-way ANOVA followed by Dunnett’s multiple comparisons test, *P < 0.05; **P < 0.01. **(F)** Statistical maps of significant CBV changes after injection of the different compounds compared to the vehicle injection (*P* < 0.05). *Z*-scores are color coded (red stands for significant increase, blue for significant decrease) and superposed on a custom fUS template which was generated by averaging realigned images of all fUS scans.

The effects of DPZ alone or in combination with MEF (i.e., combination THN201) were monitored in a coronal plane at the hippocampus level using fUS (Figures 1A,B). CBV changes were quantified in different ROI (Figure 1C). The administration of DPZ0.25 alone or MEF1 alone had limited effects on the signal compared to the baseline (Figure 1D). On the contrary, DPZ1 alone produced a persistent decrease of CBV occurring rapidly after injection in all ROI, with largest effects noted in the hippocampus and the cortex. Interestingly, in the hippocampus, the DPZ0.25 + MEF1 (THN201) combination produced hemodynamic responses different from those evoked by DPZ0.25 alone, with a decrease in CBV as large as that caused by DPZ1. A less pronounced effect was noted in the cortex, and almost none occurred in the thalamus or hypothalamus (Figure 1D). Quantitative comparison of the post-injection periods (Figure 1E) showed that the CBV responses in both DPZ1 and DPZ0.25 + MEF1 groups were significantly different from that observed after DPZ0.25 alone in the hippocampus (*P* = 0.0052 and *P* = 0.0026, respectively). Interestingly, in the latter region, whereas the non-significant trend after MEF1 or DPZ0.25 alone was toward some increase in CBV, a significant CBV decrease was noted after DPZ0.25 + MEF1 (THN201) showing that this effect did not result from additive effects of the two drugs on their own (Figure 1E). In the cortex, only DPZ1 was significantly different from DPZ0.25 (*P* = 0.048), and only a non-significant trend toward a decrease in local CBV was noted after DPZ0.25 + MEF1 (THN201) combination (Figure 1E).

A voxel-based analysis was also performed to map the significant hemodynamic effects of each treatment condition without *a priori* defined ROI (a *P*-value < 0.05 was selected as cut-off). DPZ0.25 alone induced only very discrete non-significant CBV changes (Figure 1F) whereas MEF1 alone induced clear-cut significant CBV increases in the cortex. Significant clusters were also found in the hippocampus, and to a lower extent in the thalamus (Figure 1F). On the contrary, in the cortex, the thalamus and the hippocampus, both DPZ1 alone and DPZ0.25 + MEF1 induced clear-cut bilateral CBV decreases compared to the control condition (Figure 1F).

## Discussion

The central effects of DPZ and/or MEF were investigated using the novel technique of fUS neuroimaging. fUS relies on the measurement of Power Doppler, which is proportional to CBV, at frequency higher than thousand frames per second by using plane wave transmissions (Deffieux et al., 2018). Although it is typically limited to 2D imaging, it has higher temporal resolution compared to fMRI (Tiran et al., 2017). To the best of our knowledge, our study is the first report of fUS for investigating the effects of psychoactive compounds in animal groups large enough (*n* = 8–9 mice per group) to allow statistical evaluations. DPZ at the 0.25 mg/kg dose produced only very limited, if any, changes of CBV, in line with the lack of procognitive effect of this dose that we reported recently (Droguerre et al., 2020). In contrast, DPZ at the precognitive dose of 1 mg/kg induced widespread and persistent CBV decreases. When MEF was co-administered with DPZ at low dose, a similar decreasing effect was observed, although less widespread as it appeared to be restricted to the hippocampus and some parts of the cortex. In line with our previous behavioral data (Droguerre et al., 2020), this hemodynamic pattern suggests a synergistic effect of the combination rather than possible additive effects that both drugs would produce on their own. Indeed, in the hippocampus, non-significant effects of MEF1 or DPZ0.25 alone corresponded to trends toward a CBV increase, in sharp contrast with the significant CBV decrease observed in mice injected with the THN201 combination or DPZ1 (Figure 1E). Our data with the latter drug are consistent with a previous pharmacological MRI study in rats (Hegedus et al., 2015) that reported a weak increasing effect of DPZ on the BOLD signal at low dose but a long-lasting inhibition of BOLD signal at a higher dose). Interestingly, DPZ1 and THN201-induced decreases of CBV in the hippocampus occurred in the same doses’ ranges as those producing clear-cut precognitive effects in mouse AD models (Droguerre et al., 2020). This close correlation might well be underlain through drug-induced changes in acetylcholine outflow because high concentrations of this neurotransmitter were reported to activate hippocampal inhibitory interneurons (McQuiston, 2014) and we previously observed a marked acetylcholine overflow at hippocampal level in mice administered with THN201 or DPZ1 (Droguerre et al., 2020). In support of this interpretation, a recent neuroimaging study did show that acetylcholine reduced *in vivo* neuronal activity and produced a decreased fMRI response at its injection site in brain (Zaldivar et al., 2018).

As routinely done in non-clinical neuro-imaging studies, this work has been done using anesthetized mice, constituting a limit for the interpretations, but new developments in fUS have been recently gained on awake animals (Urban et al., 2015; Tiran et al., 2017) to tackle this issue for promising further developments in pharmaco-fUS.

## Conclusion

Functional ultrasound imaging has been recently and successfully adapted to the evaluation of rodent brain activity (Mace et al., 2011). To our knowledge, our study is the first to compare the profile of two drug treatments, one marketed: DPZ, and the other under development: THN201 (DPZ + MEF), for AD using pharmaco-fUS. It points out a marked potentiating effect of MEF on DPZ in THN201, in convergence with previous studies using validated behavioral tests (to assess learning, working, and spatial memories), in the same mouse strain, after administration of both drugs in similar dose ranges (Droguerre et al., 2020). Specifically, results of the present study correlate with those of these previous studies showing the ability of MEF to (i) potentiate the effects of DPZ on cognitive performances in mice and (ii) increase DPZ-induced acetylcholine overflow in the hippocampus. More importantly, we herein describe the regional activity of THN201 in the hippocampus, the cortex and the thalamus, comparatively to DPZ alone. Among those structures which are affected in AD (Fjell et al., 2014; Aggleton et al., 2016; Setti et al., 2017), the hippocampus has been widely studied and it is now well established that hippocampal hyperexcitability can be detected years before diagnosis (Setti et al., 2017) and this region is atrophied in AD patients (Burnham et al., 2016; Josephs et al., 2017). Comparing the hemodynamic profiles of different drugs in the hippocampus, as well as other regions, using pharmaco-fUS should become of paramount importance to select the best candidates for AD, in complement of investigating brain metabolism with positron emission tomography or regional activation using functional magnetic resonance imaging (Nairne et al., 2015). More generally, pharmaco-fUS undoubtedly provides new insights in the characterization of the hemodynamic profile of drugs and constitutes an innovative non-invasive technique to move forward new drugs to development.

## Data Availability Statement

All datasets presented in this study are included in the article/supplementary material.

## Ethics Statement

The animal study was reviewed and approved by all experiments were conducted in strict accordance with the recommendations and guidelines of the European Union (Directive2010/63/EU) and strictly followed the policies of the French ethic committee for preclinical research. Procedures and protocols herein described were authorized by the French Ministry of Research (authorization reference: APAFIS19829).

## Author Contributions

BV and MV performed the fUS experiments. BV and MD analyzed the data. BV, MD, and MC designed the experiments and managed the project. MD and MC wrote the first draft. All authors reviewed the article and approved the final manuscript.

## Conflict of Interest

BV, MD, MH, FM, and MC were employed by Theranexus. This study was funded by Theranexus. The remaining authors declare that the research was conducted in the absence of any commercial or financial relationships that could be construed as a potential conflict of interest.

## Abbreviations

AD: Alzheimer’s disease
CBV: cerebral blood volume
Cx: Connexin
DPZ: donepezil
fUS: functional ultrasound imaging
MEF: mefloquine.
